# Prevalence of and factors associated with self-reported noncompliance with mandatory seatbelt-use law while driving among adults in Kuwait

**DOI:** 10.1186/s40621-020-00284-9

**Published:** 2020-11-09

**Authors:** Hashem Ridha, Fahed Bouzaber, Maryam Al-Sallal, Aisha Almutairi, Reem Al-dhubaiei, Saeed Akhtar

**Affiliations:** Department of Community Medicine & Behavioural Sciences, Faculty of Medicine, Kuwait University, PO Box 24923, 13110 Safat, Kuwait

**Keywords:** Seatbelt law, Noncompliance, Prevalence, Risk behaviors, Log-binomial model, Prevalence ratio

## Abstract

**Objectives:**

This cross-sectional study assessed the prevalence of self-reported noncompliance with mandatory seatbelt-use law and examined the factors associated with noncompliance with seatbelt-use while driving in adult working population in Kuwait.

**Methods:**

During October 2017, 822 adults aged 21–60 years from 11 government ministries and departments were enrolled in this study. Data were collected using a pre-tested, structured, and self-administered questionnaire. We computed the prevalence of self-reported noncompliance with mandatory seatbelt-use law while driving and evaluated the factors associated with noncompliance with seatbelt-use law while driving using a multivariable log-binomial regression model. The adjusted prevalence ratios (PR) and corresponding 95% confidence intervals (CI) were computed using model’s parameters’ estimates.

**Results:**

Of 822 participants, 64.4% were females, 56.6% were 21 to 30 years old, 86.5% were Kuwaitis, and 70.3% had college and/or university level education. The prevalence of self-reported noncompliance with mandatory seatbelt-use law while driving was 55.5%, whereas the prevalence of noncompliance with self-reported mandatory use seatbelt as a passenger was 80.9%. Multivariable log-binomial regression model showed that after adjusting for the influence of other variables in the model, participants were more likely to be noncompliers with mandatory seatbelt law while driving, if they believed that seatbelt does not protect against injuries during road traffic crashes (RTC) (adjusted PR = 1.20; 95% CI: 1.06–1.37; *p* = 0.004) or if they were ever fined for not wearing seatbelt (adjusted PR = 1.34; 95% CI: 1.24–1.47; *p* <  0.001). Furthermore, participants were significantly more likely to be noncompliers with mandatory seatbelt law while driving, if they were unaware of implemented mandatory seatbelt law in Kuwait (adjusted PR = 1.11; 95% CI: 1.04–1.19; *p* = 0.003).

**Conclusions:**

The prevalence of noncompliance with mandatory seatbelt-use law in the adult working population of Kuwait is considerably high. Being unaware of mandatory seatbelt use law, belief that seatbelt does not protect during RTC, and having ever been fined in the past for not having seatbelt on while driving were significant predictors of noncompliance with seatbelt-use law. These results warrant the focused mass education and rigorous enforcement of seatbelt-use law while driving. These strategies are likely to enhance the adherence to seatbelt-use law and minimize RTCs related injuries and mortality among adult drivers in this and other similar settings in the region. If implemented, future studies may look at the impact of such interventions on RTCs related frequency and severity of injuries in this and other similar settings.

## Background

Globally road traffic crashes (RTCs) represent the 8th leading cause of death for all age groups with 1.35 million people dying each year with a RTCs fatality rate (per 100,000 population) of 18.2 and cause up to 50 million injuries. Additionally, it is the leading cause of death among children and young adults aged 5–29 years (WHO 2018). The RTCs-related fatalities and injuries are disproportionally distributed worldwide with low- and middle-income countries bear the greatest burden (WHO 2018). Barring Eastern Mediterranean region, RTCs-related fatality rates (per 100,000 population) have inverse relationship with countries’ income (Nantulya and Reich 2002). For instance, across the World Health Organization (WHO) regions, RTCs-related fatality rates (per 100,000 population) are the highest in Africa (26.6) and South-East Asia (20.7) followed by Eastern Mediterranean (18.0) and Western Pacific (16.9) and lowest in Americas (15.6) Europe (9.3) (WHO 2018).

The WHO estimated RTCs-related fatality rate (per 1000,000 population) in Kuwait was 17.6 comparable with the global average rate (WHO 2018). Over the years, Kuwait implemented several interventions to minimize the RTCs and related morbidity and mortality. These interventions offered some dividend in terms of reduction in injuries and deaths (Akhtar and Ziyab 2013). Despite these efforts, between 2003 and 2009, RTCs-related 2945 deaths were recorded. Of these deaths, an overwhelming majority were males aged between 20 and 59 years (Ziyab and Akhtar 2012). With progressively rising number of vehicles on Kuwaiti roads, the number of RTCs and resulting fatalities are likely to further increase in future if present trend continues unabated. Like other Gulf Cooperation Council (GCC) countries (Abbas et al. 2011a), high-risk driving behaviours such as speeding, traffic light violation, mobile phone use, noncompliance with mandatory seatbelt law while driving in addition to host characteristics including male gender, younger age etc., are major contributors in RTCs and related injuries and fatalities in Kuwait.

RTCs are often preventable with improved driving behaviours through better road designs, drivers’ education, and strict implementation of traffic laws. Furthermore, in the event of RTCs, a substantial reduction in injuries and/or deaths rates can be attained by taking appropriate vehicle crash safety measures (WHO 2018). Reportedly seatbelt use both by drivers and passengers reduces morbidity and mortality. In fact, in the event of a RTC, seatbelt use attenuates the severity of injuries and contributes to save lives (Hodson-Walker 1970). According to an estimate, up to 80% of all RTC-related deaths can be prevented by proper seatbelt use (Cummings 2002). The WHO estimated at least 40% reduction in the risk of severe injuries among seatbelt users both among drivers and front seat passengers during RTCs (WHO 2015). Even with current generation of airbags, seatbelts reduce the risk of almost all injuries (Kuk and Shkrum 2018). Despite these obvious and tangible public health advantages, seatbelt use while driving is still very low (48%) in GCC countries (Abbas et al. 2011a), even lower (27.2%) in Saudi Arabia (Bendak 2007), and other countries including Iran (33%) (Mohammadi 2011), located in WHO Eastern Mediterranean Region as oppose to developed countries (Bendak 2005). Furthermore, a very high proportion of drivers use the seatbelt while driving in USA (87%), UK (90%), Australia (97%) as compared to high income developing GCC countries including Qatar (73%) (Mahfoud et al. 2015), Kuwait (42%) (Raman et al. 2014), UAE (29%) (Barss et al. 2008) and Bahrain (20%) (WHO 2015).

Noncompliance with mandatory seatbelt-use law is a significant contributory factor in occupants’ injuries and/ or death rates (Abbas et al. 2011b). In Kuwait, mandatory seatbelt use law for drivers was enacted 1976 and for all front seat passengers in 1994 (Raman et al. 2014; Koushki and Bustan 2006). Despite the implementation of seatbelt law while driving, over the years the prevalence of noncompliance among the drivers remained nearly constant as 45% in 1996 (Koushki et al. 1996), and 42% in 2014 (Raman et al. 2014). However, in-depth evaluation of the factors associated with this high prevalence of noncompliance with seatbelt law have not been carried out. Furthermore, Raman and colleagues focused only on the general driving behaviours of the participants in relation to seatbelt use and did not assess the unawareness about enacted mandatory seatbelt law, snags in using seatbelt and perceived effectiveness of seatbelt related protection against severe injuries in an event of a RTC etc., (Raman et al. 2014). These deficits in the knowledge and understanding of the driving population about the effectiveness of seatbelt use might have been contributing in the high prevalence of noncompliance with seatbelt-use law while driving. Therefore, this cross-sectional study was designed to i) assess the prevalence of noncompliance with mandatory seatbelt-use law in the working adult population in Kuwait, and ii) identify the factors associated with noncompliance to seatbelt-use law in this population with focus on realism of seatbelt use while driving.

## Participants and methods

### Study design, setting and population

During October 2017, this cross-sectional study was conducted to assess the prevalence of noncompliance with mandatory seatbelt-use law in the working adult population in Kuwait, and identify the factors associated with noncompliance to seatbelt-use law in this population. The target population was working adults aged 21–60 years (49% of the country total population), both males and females of any nationality employed at government ministries and departments from all the six governorates of the country. The rationale for choosing the target population in this age bracket was that they constitute the country’s workforce, mostly carry driving license and drive almost daily. We selected 11 ministries as a sample of convenience because of their location in ministerial complex which is easily approachable during working hours.

### Questionnaire development and its pre-testing

The questionnaire was developed in both Arabic and English. The questionnaire comprised 21 questions grouped into two sections. Section 1 contained closed-ended questions on participant’s sociodemographic information including age, gender, nationality, marital status, education level and monthly income (Kuwait dinars, KD). In section 2, the participants were enquired about their seatbelt use practice both as a driver and passenger separately in two questions i.e. how often do, they use the seatbelt as a driver (and passenger). Both the questions were asked on Likert type scale (i.e. always, nearly always, sometimes, rarely, never, don’t know); if they were regular user of seatbelt, and if not, we asked them about the reasons. Additionally, we asked them if they were ever fined for not using the seatbelt, and if they think that seatbelts protect drivers from RTC related injuries and fatalities. The questionnaire was reviewed by two faculty members and some of the questions were revised in the light of comments on the questionnaire. The questionnaire was pre-tested on 10 public-sector employees like our target population. Based on the pre-testing, the questionnaire was further improved to enhance its readability by the respondents. On average it took about eight to 10 min to complete the questionnaire. An informed consent was obtained from all the participants before handing over the questionnaire for completion.

### Sampling, data collection and sample size

As noted earlier, population of interest in this study was Kuwaiti residents working population ages between 21 and 60 years. We enrolled our study participants from various Government ministries including Finance, Oil and Petroleum, Commerce and Industry, Foreign affairs, and Interior. On the day of data collection, we visited the given ministries and the participants were enrolled as a sample of convenience. We approached the participants during their break time, we explained the objectives of the study and requested for their willingness to participate in the study. Consenting participants were handed over the questionnaire for completion. We tried to check the filled questionnaire at the spot to see whether all the questions were answered.

To address both the study objectives, sample size was computed in two phases. First, for estimation of prevalence of noncompliance with seatbelt-use law, we needed 384 participants to estimate an assumed 50% prevalence of noncompliance with mandatory seatbelt-use law in the target population at a 95% confidence level with 5% bound on error of estimation. Second, to identify the potential risk factors for noncompliance with mandatory seatbelt-use law, with a minimum prevalence ratio (PR) of 2.0 at 5% significance level with a study power of 90%, we needed 354 noncompliers and 354 compliers with mandatory seatbelt-use law assuming a prevalence of unawareness of seatbelt-use law as 25 and 15% among noncompliers and compliers respectively (Fleiss et al. 2013). To account for potential refusals, we inflated the final sample size to 400 participants in each of the study groups.

### Ethics

The study protocol and data collection instrument were approved by the institutional ethics committee for student’s research. As noted above, to the participants, we explained the study objectives before asking for their written informed consent. We used self-administered anonymous questionnaire that carried no identification of the participants other than the serial number. Administrative permission was sought from each ministry for the enrollment of study participants.

### Data analysis

We computed the descriptive statistics using the proportion and percentages. Responses to seatbelt compliance initially recorded on Likert scale (i.e. always, often, sometimes, rarely never, don’t know) both as a driver and as a passenger were each merged into two categories i.e. compliance with seatbelt-use law (coded as 0, if the participant reportedly always used the seatbelt) or noncompliance with seatbelt law (coded as 1, if the participant reportedly used seatbelt less frequently than always). This dichotomous variable of noncompliance with mandatory seatbelt-use law while driving was used as a dependent variable in subsequent analyses. The main rationale for collapsing five categories of the response variable to transform it as a binary variable was to make envisage comparison between two groups, i.e., fully seatbelt-law abiding participants with those with less than optimal law observing participants. Chi-squared analysis was conducted to evaluate the significance of association between dichotomous outcome variable (i.e. noncompliance status as defined above) and demographic and driving related variables. Since, odds ratio overestimates the association of predictors with common outcomes, we used unadjusted and adjusted PR as a measure of association between the independent variables and the study outcome estimated using log-binomial models (Altman et al. 1998). PRs are estimated from the coefficients of log-binomial regression model or Poisson regression model with robust variance. Under correct model specification, the log-binomial regression model and Poisson regression model with robust variance have been shown to yield comparable point estimates and standard errors (Chen et al. 2018). Presumably in this study, the relationship between self-reported noncompliance and potential predictors conformed to the functional form specified in log-binomial model and that all the known confounding variables were correctly measured and accounted for, thus met the model assumptions. Univariable log-binomial regression model was used to quantify the magnitude of unadjusted association of each of the categorical variables with outcome. The variables significantly (*p* ≤ 0.150) related with the outcome variable in univariable analyses were considered for inclusion in multivariable analysis. Backward stepwise procedure was used to select the final multivariable log-binomial regression model. Regardless of the statistical significance, age, gender, and nationality were retained in the model to adjust for their potential confounding effects. The final multivariable log-binomial regression model comprised variables which were independently and significantly (*p* <  0.05) related to noncompliance to mandatory seatbelt-use law status. Adjusted PR and their corresponding 95% confidence intervals (CI) were used for interpretation of the final model.

## Results

### Description of the study sample

We invited 850 working adults to participate in the study, 822 consented (response rate = 96.7%) and completed the questionnaires. Of the participants, 56.6% were between 21 and 30 years of age. Majority (64.4%) of the participants were female, Kuwaiti nationals (86.5%) and had college and/or university level education (70.3%). The distributions of marital status, monthly income (KDs) and governorate of residence are in Table 1.

**Table 1:** Sociodemographic characteristics of participants enrolled in a cross-sectional study of prevalence of noncompliance with mandatory seatbelt-use law while driving in Kuwait, October 2017 (*N* = 822)

Demographic characteristics	n	%
Age (years)^a^		
21–30	465	56.6
31–40	240	29.2
> 40	116	14.1
Gender		
Male	293	35.6
Female	529	64.4
Nationality		
Kuwaiti	711	86.5
Non-Kuwaiti	111	13.5
Governorate		
Alasimah	240	29.2
Hawalli	221	26.9
Farwaniya	144	17.5
Mubarak Alkabeer	117	14.2
Ahmadi	48	5.8
Jahra	52	6.3
Marital status^a^		
Never married	431	52.5
Ever married	390	47.5
Education level		
Less than high school	24	2.9
High school	161	19.6
Collage/university degree	578	70.3
Higher education	59	7.2
Total monthly income (Kuwaiti dinar)^a^		
< 1000	232	28.3
1000–2000	325	39.6
2001–3000	134	16.3
> 3000	129	15.7

^a^Categories’ total may not add to 822 due to missing data

### Prevalence of noncompliance with mandatory seatbelt-use law

The prevalence of noncompliance with mandatory seatbelt-use law while driving was 55.5% (456/ 821), whereas the prevalence of noncompliance with mandatory seatbelt law while riding a vehicle as a passenger was 81.1% (665/ 820) in our sampled population. Of 299 participants who responded to a multiple-choice question on reasons for not using the seatbelt while driving, 180 (60.3%) participants cited discomfort in using seatbelt (Table 2).

**Table 2:** Prevalence of noncompliance with mandatory seatbelt-use law while driving in adult working population enrolled in a cross-sectional study, Kuwait, October 2017 (*N* = 822)

Driving-related variables	n	%
Seatbelt use while driving^a^ (multiple options question)		
Always	365	44.5
Often	154	18.8
Sometimes	158	19.2
Rarely	71	8.6
Never	69	8.4
Don’t know	4	0.5
Seatbelt use while driving^a^ (binary)		
Noncompliance (irregular use)	456	55.5
Compliance (always use)	365	44.5
Seatbelt use when riding a car as a passenger^a^		
Always	155	18.9
Often	132	16.2
Sometimes	148	18.2
Rarely	125	15.4
Never	253	31.1
Don’t know	7	0.9
Seatbelt use as a passenger^a^ (multiple options question)		
Noncompliance (irregular use)	665	81.1
Compliance (always use)	155	18.9
Reasons for using seatbelt while driving among participants who reportedly always use seatbelt		
For my own safety	316	86.6
Afraid of the law enforcement personnel	23	6.3
Most of my family members wear it	1	0.3
Others	25	6.8
Reasons for not using seatbelt^a^ while driving among participants who reportedly irregularly use seatbelt		
Seat-belt use law is not enforced	55	18.4
Seatbelt is useless	17	5.7
My driving skills are excellent	13	4.3
Using seatbelt is uncomfortable	180	60.2
Others	34	11.4

^a^ Categories total may not add to 822 due to missing data

### Chi-squared analysis of association between demographics, driving-related variables and noncompliance with mandatory seatbelt-use law while driving

Chi-squared analysis showed significant (*p* <  0.150) association between age, gender, nationality, marital status, governorate of residence and noncompliance with seatbelt-use (Table 3). Similarly, most of the driving-related variables including previous involvement in RTC, belief that seatbelt provides protection against injuries during RTC, ever fined for not using seatbelt while driving, unawareness of mandatory seatbelt-use law in Kuwait, whether or not seatbelt law is effectively enforced and mobile phone use while driving were significantly (*p* <  0.150) associated with noncompliance of mandatory seatbelt law on chi-squared analysis. Additionally, we compared men and women for reasons for not wearing the seatbelt; more women than men responded that ‘seatbelt-use law is not enforced in Kuwait’ (54.5% vs. 45.5%), ‘seatbelt is useless’ (64.7% vs. 35.3%), ‘my driving skills are excellent’ (53.8% vs. 46.2%), ‘seatbelt is uncomfortable’ (65.6% vs. 34.4%) and other reasons (41.2% vs. 58.8%). The differences across these proportions were statistically marginally significant (*p* = 0.078) (Table 4).

**Table 3:** Chi-squared analysis of the relationship between demographic characteristics and noncompliance with mandatory seatbelt-use law while driving in adult working population, Kuwait, October 2017. (*N* = 822)

Characteristic	Category total	Noncompliance with seatbelt-use law (*n* = 456; 55.5%)	*P*-value
		n (%)	
Age			0.042
21–30	465	249 (53.5)	
31–40	240	149 (62.1)	
> 40	116	58 (50.0)	
Gender			0.011
Male	293	180 (61.4)	
Female	528	276 (52.3)	
Nationality			0.017
Kuwaiti	710	406 (57.2)	
Non-Kuwaiti	111	50 (45.0)	
Governorate			0.074
Alasimah	239	137 (57.3)	
Hawalli	221	105 (47.5)	
Farwaniya	144	81 (56.3)	
Mubarak Alkabeer	117	68 (58.1)	
Ahmadi	48	31 (64.6)	
Jahra	52	34 (65.4)	
Marital status			0.076
Never married	430	226 (52.6)	
Ever married	390	229 (58.7)	
Educational level			0.304
Less than high school	24	12 (50.0)	
High school	161	95 (59.0)	
Collage/university degree	577	322 (55.8)	
Higher education	59	27 (45.8)	
Total monthly income (KD)			0.521
< 1000	232	117 (50.4)	
1000–2000	324	188 (58.0)	
2001–3000	134	83 (61.9)	
> 3000	129	66 (51.2)

**Table 4:** Chi-squared analysis of relationship between driving-related variables and noncompliance with mandatory seatbelt-use law while driving in adult working population, Kuwait, October 2017. (*N* = 822)

Variable	Category total	Noncompliance with seatbelt-use law (*n* = 456; 55.5%),	*p*-value
		n (%)	
Involved in RTC			0.133
Yes	261	135 (51.7)	
No	560	321 (57.3)	
Believe seatbelt protects from injuries during RTC			< 0.001
Yes	762	410 (53.8)	
No	58	46 (79.3)	
Ever been fined for not using seatbelt			< 0.001
Yes	195	157 (80.5)	
No	620	298 (48.1)	
Does Kuwait have mandatory seatbelt use law?			0.001
Yes	522	268 (51.3)	
No	299	188 (62.9)	
Is law regarding seatbelt effectively enforced?			0.046
Yes	194	89 (45.9)	
No	326	179 (54.9)	
While driving, do you usually speed over limit?			0.322
Always	53	32 (60.4)	
Often	106	60 (56.6)	
Sometimes	261	156 (59.8)	
Rarely	199	99 (49.7)	
Never	172	90 (52.3)	
Don’t know	7	4 (57.1)	
Do you use mobile phone while driving?			0.09
No	121	51 (42.1)	
Yes	699	405 (57.9)	
What government should do to increase seatbelt-use?			0.624
Raise awareness	399	225 (56.4)	
Educational programs for children	61	36 (59.0)	
Increase the penalty for not Wearing seatbelt	315	167 (53.0)	
Other	46	28 (60.9)	
Participants’ proposed penalty for not wearing seatbelt			0.001
No penalty	58	43 (74.1)	
Imposition of heavy fine	400	230 (57.5)	
Confiscation of driving license	170	82 (48.2)	
Confiscate your car	87	50 (57.5)	
1 to 30 days in prison	56	20 (35.7)	
Other	43	26 (60.5)	

### Multivariable log-binomial regression model

Final multivariable log-binomial regression model showed that after adjusting for the influence of other variables in the model, participants were more likely to be noncompliers with mandatory seatbelt law while driving, if they believed that seatbelt does not protect from injuries during RTC (adjusted PR = 1.20; 95% CI: 1.06–1.37; *p* = 0.004) or if they were ever fined for not wearing seatbelt (adjusted PR = 1.34; 95% CI: 1.24–1.47; *p* <  0.001). Furthermore, participants were significantly more likely to be noncompliers with mandatory seatbelt law while driving, if they were unaware of implemented mandatory seatbelt law in Kuwait (adjusted PR = 1.11; 95% CI: 1.04–1.19; *p* = 0.003) (Table 5).

**Table 5:** Multivariable log-binomial regression model^a^ of factors associated with self-reported noncompliance with mandatory seatbelt-use law assessed in a cross-sectional study of adult working population. Kuwait, October 2017

Variable	Unadjusted PR^a^ (95% CI), *p*-value	Adjusted PR (95% CI)	*P*-value**
Do you think seatbelt protects from injuries during road traffic crash? (no vs. yes)	1.29 (1.13–1.47)	1.20 (1.06–1.37)	0.004
Have you ever been fined for not wearing seatbelt? (yes vs.no)	1.38 (1.28–1.49)	1.34 (1.24–1.45)	< 0.001
Do you think Kuwait has a mandatory seatbelt use law? (no vs. yes)	1.12 (1.05–1.20)	1.11 (1.04–1.19)	0.003
Age (completed years)			
21–30	Ref	Ref	–
31–40	1.09 (1.01–1.18)	1.07 (1.00–1.16)	0.059
41–50	1.00 (0.89–1.12)	1.01 (0.90–1.13)	0.860
> 50	0.87 (0.73–1.05)	0.87 (0.73–1.04)	0.123
Gender (female vs. male)	0.91 (0.85–0.98)	0.92 (0.86–0.99)	0.032
Nationality (Kuwaiti vs. Non-Kuwaiti)	1.13 (1.02–1.25)	1.10 (0.99–1.21)	0.070

*PR* Prevalence ratio, *CI* Confidence interval

** *p*-value associated with adjusted PR

^a^ Model was adjusted for age, gender and nationality effects and were retained in the final model regardless of statistical significance

## Discussion

### Prevalence of noncompliance with mandatory seatbelt-use law

WHO estimates that seatbelt use while driving reduces up to 40% risk of severe injuries in the event of RTC, yet the level of seatbelt use in the WHO Eastern Meditation region is very low (WHO 2015). This study assessed the prevalence of noncompliance with mandatory seatbelt-use law while driving and examined the demographic and driving-related factors associated with noncompliance to mandatory seatbelt-use law while driving in the adult working population in Kuwait.

In this study, the prevalence of noncompliance with mandatory seatbelt-use law while driving was 55.5% (456/821), and the prevalence of noncompliance with seatbelt-use law while riding a vehicle as a passenger was 80.9% (658/ 813) in the adult working population of Kuwait. The estimated 55.5% noncompliance with mandatory seatbelt-use law while driving in this study is higher than a corresponding figure of 42% reported by an earlier study conducted in Kuwait (Raman et al. 2014). The difference in two estimates from this and previous study possibly could be due to the sampled populations. Our study sample predominantly comprised women (64.4%) compared with the corresponding figure (43.9%) reported previously (Raman et al. 2014). Furthermore, compared with men, women are known as relatively more safe drivers (Laapotti et al. 2003). Furthermore, in this study, we found more men (61.4%) than women (52.3%) as noncompliers with mandatory seatbelt-use law while driving.

The 55.5% prevalence of noncompliance with mandatory seatbelt-use law while driving in our study was lower than the estimates from some neighboring countries including UAE (71%) and Bahrain (80%) (WHO 2015; Raman et al. 2014). However, the estimate of noncompliance with mandatory seatbelt-use law sought in this study (55.5%) was substantially higher than 27% reported from Qatar (Mahfoud et al. 2015). Furthermore, prevalence of noncompliance with mandatory seatbelt-use law in this study was much higher than the figures reported among adult driving populations from high income developed countries including USA (13%), UK (10%), and Australia (3%) (Mahfoud et al. 2015). This difference in the proportions of noncompliance in this and cited studies is ostensibly due to population awareness of potential benefits of seatbelt use and relative rigor of mandatory seatbelt law implementation by respective traffic laws enforcement agencies. Additionally, in this setting, it may well be that the amount of fine imposed on seatbelt law violators is very low. Resultantly, mere seatbelt law enactment was not delivering the desired results. Therefore, targeted education about the traffic laws followed by strict implementation and inflicting heavy penalty to seatbelt law violators may help minimize the related morbidity and mortality in this and other settings in the region as happened relatively recently in adult driving population in Saudi Arabia, wherein strict enforcement of law and heavy penalties to seatbelt law violators resulted to an increased seatbelt use from 34 to 76% among drivers (Alghnam et al. 2018a).

### Factors associated with noncompliance to mandatory seatbelt-use law

In this study, we found that the study participants were more likely to be noncompliers with mandatory seatbelt use law, if they held the belief that seatbelts did not protect in the event of a RTC. A similar gap in the understanding of seatbelt usefulness has been demonstrated among Turkish drivers (Simşekoğlu and Lajunen 2008). Campaigns for public education on traffic laws specifically on potential benefits of seatbelt use should be considered.

The participants were more likely to be noncompliers with mandatory seatbelt law, if they have been fined in the past for not having a seatbelt on while driving. This probably shows the ineffectiveness of the current penalty system in preventing drivers from violating the traffic laws including seatbelt-use law while driving. From Turkey, a study reported that seatbelt-use noncompliance was associated with disobeying traffic laws (Simşekoğlu and Lajunen 2008). It also indicates that drivers who disregard the traffic laws are more likely to disregard seatbelt-use law as well. For that matter, an educational campaign focused on traffic laws including that of seatbelt-use is likely to improve the current situation as it transpired elsewhere (Shults et al. 2004). Furthermore, the extent of penalty for noncompliance with seatbelt law ought to be reexamined considering the high proportion of (80.5%) the participants who continued to be noncompliers with seatbelt law despite having been fined in the past for violating this law.

This study found that a considerable proportion of the study participants (36.4%), were unaware of the mandatory seatbelt-use law in Kuwait. Unsurprisingly, the participants who were unaware of the mandatory seatbelt-use law while driving were more likely to be noncompliant with seatbelt-use law. A study in Pakistan found comparable results (Klair and Arfan 2014). It appears that traffic laws including mandatory use of seatbelt while driving seem to be loosely imposed on driving population in Kuwait. High prevalence of noncompliance with these traffic laws warrants the need for rigorous educational campaigns followed by strict enforcement of traffic laws including that of mandatory seatbelt law and heavy fines to noncompliers. Such interventions have paid dividends in a neighboring Saudi Arabia (Abbas et al. 2011a), Iran (Mohammadi 2011) and are likely to contribute in minimizing violation of seatbelt law and RTC-related severe injuries in this and other similar settings.

In this study, the mobile phone use while driving was statistically non-significant for retaining in the final multivariable log-binomial model. However, the participants who (in violation of law on mobile use while driving), reportedly always (*n* = 63) or nearly always (*n* = 115) used mobile phone while driving, respectively 71.4 and 65.7% of them violated the seatbelt-use law as well. Incidentally in two neighboring countries in the region including Qatar and Saudi Arabia, dual violation of seatbelt law and mobile phone use while driving was commonly observed (Mahfoud et al. 2015; Alghnam et al. 2018b). Such drivers with dual violation of laws not only endanger their lives but also put at risk the lives of other motorists and passengers on the road. Concerted efforts are needed to run awareness campaigns and stern law enforcement. These interventions are likely to curb such violations and improve compliance with laws requiring seatbelt use and prohibiting mobile phone use while driving.

To a question about the reasons for not wearing seatbelt while driving, the participants have had mixed responses. Furthermore, we compared men and women for reasons for not wearing the seatbelt and found that more women than men responded that ‘seatbelt law is not enforced in Kuwait’, ‘seatbelt is useless’, ‘my driving skills are excellent’ and ‘seatbelt is uncomfortable’. A point worth noting is that about 66% women reported the reason for not using seatbelt is discomfort. This could possibly be owing to a women’s smaller stature that leads to poorer fit of seatbelts which may rub the neck if the vehicle does not have an adjustment and/ or the origin of the asserted discomfort could be related to wearing of the hijab or niqab or even the man’s head dress. The difference across these proportions were only marginally significant on univariable analysis and could not qualify for inclusion the final multivariable model. Future studies my look into the participants reported reasons more objectively.

### Study strengths and limitations

The present study has few notable strengths including; i) This study confirmed that noncompliance with seatbelt use law continued to be very high (55.5%); ii) As opposed to acclaimed continuing efforts by traffic laws enforcement agency, this study unraveled a substantial increase in the prevalence of noncompliance with seatbelt-use law compared to a previously reported estimate in Kuwait (42%) (Raman et al. 2014); iii) This study disentangled two previously unknown factors that were associated with the prevailing virtually constant prevalence of noncompliance with seatbelt use law in Kuwait including; *first,* unawareness about the decreed seatbelt law in the country, and *second*, knowledge deficit about the effectiveness of seatbelt in protecting against injuries in the event of a RTC.

Some limitations of this study need be taken into consideration while interpreting its results: i) As with all cross-sectional studies, casual inferences are limited from these data. ii) Self-reports might have slightly underestimated the noncompliance with mandatory seatbelt-use law, yet the prevalence estimate of noncompliance undesirably was still very high; iii) The sample may not be truly representative of the Kuwaiti population considering the overrepresentation of female and Kuwaiti nationals compared to the general Kuwaiti population. This suggests a selection bias in the study sample and seems to have happened because, apparently Kuwaiti females outnumber the Kuwaiti men in ministries included in this study. Therefore, female predominance in our sample might have resulted in underestimation of the noncompliance of seatbelt use law because females generally are regarded as having better driving behaviours (Koushki and Bustan 2006; Al-Balbissi 2003). Having noted this, noncompliance to seatbelt law was still very high in the study sample; iv) In addition, the study was limited to governmental facilities. Thus, private companies and other sectors are not represented in the sample. The private companies incline to employ non-Kuwaiti, who generally are reluctant to violate seatbelt-use law, resultantly overestimation of noncompliance with seatbelt-use law in the study sample. Future studies may consider strategies to draw a representative sample; v) The study focused solely on the adult working population of Kuwait which excluded everyone below the age of 21 years. This might have slightly influenced the study as the younger population tends to be more irregular users of seatbelts compared to adults (Raman et al. 2014); vi) Finally, some variables had missing data with a likelihood of introduction of information bias in various parameters’ estimates in study. The missing data points might have resulted because the participants could not recall in completing their answers to those questions. We hope the level of this bias was minimal and had negligible impact on parameters’ estimates in the study.

## Conclusion

The prevalence of noncompliance with mandatory seatbelt-use law in the adult working population of Kuwait is considerably high in the backdrop of seatbelt law that was decreed more than two decades ago. Being unaware of mandatory seatbelt-use law in the country, belief that seatbelt does not protect during RTC, and having ever been fined in the past for not having seatbelt on while driving were significant predictors of noncompliance with mandatory seatbelt-use law. Therefore, a focused educational campaign and rigorous enforcement of seatbelt-use law may bring substantial improvement in the adherence to this law and minimize the RTCs related injuries and mortality among the adult drivers in Kuwait as much these interventions have shown their effectiveness in the neighboring countries in the region including Iran and Saudi Arabia, where in particular seatbelt use increased from 34 to 76% subsequent to intervention (Alghnam et al. 2018a; Soori et al. 2009, 2011). If implemented, future studies may look at the impact of such interventions on RTCs related frequency and severity of injuries in this and other similar settings.

## Data Availability

The dataset analyzed during the current study is available from the corresponding author on reasonable request.

## Abbreviations

RTC: Road traffic crash
GCC: Gulf Cooperation Council
PR: Prevalence ratio
CI: Confidence interval
WHO: World Health Organization
KD: Kuwaiti Dinars
